# Aripiprazole for the treatment of tic disorders in children: a systematic review and meta-analysis

**DOI:** 10.1186/s12888-015-0504-z

**Published:** 2015-07-29

**Authors:** Chun-Song Yang, Hong Huang, Ling-Li Zhang, Cai-Rong Zhu, Qin Guo

**Affiliations:** Department of Pharmacy, Evidence-based Pharmacy Center, West China second hospital, Key Laboratory of Birth Defects and Related Diseases of Women and Children, Sichuan University, Chengdu, China; West China School of Public Health, Sichuan University, Chengdu, China; Department of Pediatrics, West China second hospital, Key Laboratory of Birth Defects and Related Diseases of Women and Children, Sichuan University, Chengdu, China; West China Second University Hospital, Sichuan University, No.20,Third Section, Renmin NanLu, Chengdu, Sichuan 610041 People’s Republic of China

**Keywords:** Aripiprazole, Tic disorders, Children, Systematic review

## Abstract

**Background:**

Tic disorders (TDs) are common neuropsychiatric disorders in children. Typical antipsychotics, such as haloperidol and pimozide have been prescribed to control tic symptoms as first-line agents. However, adverse effects have led to the use of newer atypical antipsychotics. Aripiprazole is one of alternatives. The aim of this study was to evaluate the efficacy and safety of aripiprazole for children with TDs.

**Methods:**

Randomized controlled trials (RCTs), quasi-RCTs and control studies evaluating aripiprazole for children with tic disorders were identified from PubMed, Embase, Cochrane library, Cochrane Central, four Chinese database and relevant reference lists. Quality assessment referred to the Cochrane Handbook for Systematic Reviews of Interventions.

**Results:**

Twelve studies involving 935 participants were included. The general quality of included studies was poor. Only one study used placebo as a control and others used positive drug controls. Participants were aged between 4 and 18 years. The period of treatment ranged from 8 to 12 weeks. Seven studies (*N* = 600 patients) used the YGTSS scale as the outcome measurement, and there was no significant difference in reduction of the total YGTSS score between the aripiprazole and positive control groups (MD = −0.48, 95 % CI [−6.22, 5.26], *P* = 0.87, I^2^ = 87 %). Meta-analysis of four of the studies (*N* = 285 patients) that compared aripiprazole with haloperidol showed that there was no significant difference in reduction of the total YGTSS score (MD = 2.50, 95 % CI [−6.93, 11.92], *P* = 0.60, I^2^ = 88 %). Meta-analysis of two studies (*N* = 255 patients) that compared aripiprazole with tiapride showed that there was no significant difference in reduction of the total YGTSS score (MD = −3.15, 95 % CI [−11.38, 5.09], *P* = 0.45, I^2^ = 86 %). Adverse events (AEs) were reported in 11 studies. Drowsiness (5.1 %–58.1 %), increased appetite (3.2 %–25.8 %), nausea (2 %–18.8 %) and headache (2 %–16.1 %) were common AEs.

**Conclusion:**

In conclusion, aripiprazole appears to be a promising therapy for children with TDs. Further well-conducted RCTs are required to confirm this issue.

## Background

Tic disorders (TDs) are common neuropsychiatric disorders in children. These disorders are characterized by sudden, fast, repetitive, non-rhythmic, and stereotyped motor movements and/or phonic production [1]. TDs are often classified as transient tic disorder (TTD), chronic tic disorder (CTD), and Tourette syndrome (TS). Symptoms of common comorbidities of TDs (*i.e.*, attention-deficit hyperactivity disorder [ADHD], obsessive-compulsive disorder, oppositional defiant disorder, and other mood disorders) often co-exist [2]. A meta-analysis of the prevalence of TDs showed that the prevalence of TS is 0.77 % (95 % confidence interval [CI]: 0.39–1.51), TTD is the most common TD, with a prevalence of 2.99 % (95 % CI: 1.60–5.61), and CTD has a prevalence of 1.61 % (95 % CI: 0.92–2.83) [3].

Currently, pharmacotherapy is the main management for motor/vocal tics and comorbidity symptoms. Typical antipsychotics, such as haloperidol and pimozide have been prescribed to control tic symptoms as first-line agents [4]. However, adverse effects, such as acute dystonic reactions, akathisia, tardive dyskinesia, extrapyramidal syndrome, and prolonged QTc, are problematic and have led to the use of newer atypical antipsychotics [5]. Among the atypical antipsychotics, the use of risperidone and aripiprazole for the treatment of TS has been described in randomized controlled trials (RCTs) and case series studies [6–12]. In 2013, a 14-week prospective open-label study [11] evaluated the efficacy of aripiprazole for TDs in patients who were aged between 4 and 18 years, including 81 children. This previous study showed that the mean reduction in the motor tic score was 51.0 %, that in the vocal tic score was 67.1 %, and that in the total Yale Global Tic Severity Scale (YGTSS) score was 70 % after treatment. The authors concluded that aripiprazole is effective for the short-term treatment of TD, especially vocal tics, with few adverse effects. In 2012, another 12-week open-label study [12] assessed the use of aripiprazole in a consecutive group of 28 patients with the primary diagnosis of TS and co-morbid ADHD, a combined subtype. This previous study showed that the YGTSS score and the ADHD rating scale IV significantly improved (*p* < 0.001) after treatment. With regard to the YGTSS, there was a reduction of 42.5 % in motor tics, 47.9 % in vocal tics, and 32.3 % in the tic impairment. A total of 67.9 % of patients had a reduction of at least 50 % of the total YGTSS score [12]. Additionally the results of two RCTs that were recently published showed that aripiprazole is efficacious, generally tolerated, and safe for TDs [9, 10].

Aripiprazole has been used to treat schizophrenia and bipolar disorder for adults and adolescents for many years. The mechanisms of action may be a partial agonist on D_2_ and 5-hydroxytryptamine 1A receptors and an antagonist on 5-hydroxytryptamine 2A receptors [13]. Aripiprazole may also have beneficial effects in patients with TDs with a lower risk of side effects than other atypical neuroleptics [14].

Ghanizadeh’s study [15] systematically reviewed the efficacy and safety of aripiprazole for children with TDs. They concluded that aripiprazole is effective for treating tic disorders including TS in children and adolescents. Additionally, aripiprazole’s adverse effect profile is safer than pimozide and some other antipsychotics. However, the included studies in this systematic review have several limitations, including a small sample size, an open-label approach, being case reports or case series, and the short-term nature of the studies. Recently, two RCTs that evaluated the efficacy of aripiprazole for children with TDs were published [9, 10]. Many studies have also been published in China. Therefore, this systematic review [15] may have missed some important information. In addition, the newer atypical antipsychotics can reduce the risk of extrapyramidal symptoms. However, atypical antipsychotics are associated with metabolic side effects, including weight gain and hyperlipidemia (an abnormally high concentration of fatty substances in the blood) [16]. Therefore, the evidence for the efficacy and safety of aripiprazole for children with TD needs to be updated.

## Methods

### Inclusion and exclusion criteria

#### Types of studies

All RCTs, quasi-RCTs, and open-label control studies comparing aripiprazole with placebo or other drug(s) used in the treatment of children with TDs were included. Trials were excluded if (1) the data for children could not be obtained (even though we attempted to contact the original study investigators), and (2) they compared different doses of drugs (*i.e.*, the treatment group used high [or low] doses of aripiprazole and the control group used low [or high] doses).

#### Types of participants

Patients with the clinical diagnosis of a TD were included. The widely used definitions of TDs are in the following guidelines: (1) the *Diagnostic and Statistical Manual of Mental Disorders*-III (DSM-III), DSM-IV, or DSM-IV-Text Revision [17–19]; (2) the *International Classification of Diseases*-10 (ICD-10) [20]; and (3) the *Chinese Classification and Diagnostic Criteria of Mental Disorders* (CCMD) [21]. The age of participants was younger than 18 years.

#### Types of interventions

All RCTs, quasi-RCTs, and open-label control studies that administered aripiprazole used either alone or as an add-on to an approved treatment for TDs were included. Comparisons included (1) aripiprazole versus placebo only, (2) aripiprazole plus approved treatments versus placebo plus approved treatments, and (3) aripiprazole versus approved treatments (*i.e.*, haloperidol and tiapride).

### Types of outcome measurements

#### Primary outcomes

We included studies that measured outcomes using one of the following scales or methods: (1) the YGTSS [22]; (2) the Clinical Global Impression (CGI) Scale [23]; (3) the Tourette Syndrome Global Scale [18]; (4) the Tourette Syndrome Symptom List [18]; (5) the Clinical Global Impression Tic Severity Scale [19]; and (6) the Tourette Syndrome Severity Scale [23].

#### Secondary outcomes

The secondary outcomes included improvement of tic symptoms that were assessed by authors’ self-definition and adverse events (AEs), which were measured using the following scales or methods: (1) the CGI Scale, Adverse Events [23], (2) the Abnormal Involuntary Movement Scale, (3) the Extrapyramidal Symptom Rating Scale, (4) weight gain, (5) abnormalities or changes on an electrocardiogram [18], and (6) other reported AEs.

### Search strategy

Two reviewers (Yang and Huang) independently identified studies through searches of PubMed (1966–2014.6), EMBASE (1974–2014, Issue 6), the Cochrane Library (2014, Issue 6), Cochrane Controlled Trials databases (CENTRAL 6, 2014), the Chinese Biomedical Literature Database (CBM, 1978–2014.6), China National Knowledge Infrastructure (CNKI, 1980–2014.6), the Chinese Science and Technique Journals Database (VIP, 1989–2014.6), the Wanfang Database (http://www.wanfangdata.com/) (1990–2014.6), and reference lists of relevant articles. The terms “aripiprazole”, “tourette syndrome”, “tic disorders”, and “tics” were combined using “and” or “or” for searching for relevant studies. The search was restricted to human studies and the language of publications was restricted to English or Chinese.

### Selection of studies and data extraction

Two reviewers (Yang and Huang) independently screened the titles and abstracts of every record. Full articles were obtained when either information provided in the title or abstracts conformed to the selection criteria outlined previously, or could not be ascertained because of limited information. To include studies, data were independently extracted by each reviewer and entered into a standardized form. The data extraction form included the following contents: (1) general characteristics of studies, (2) the general characteristics of patients, (3) the diagnostic criteria, (4) sample size, (5) comparisons, (6) outcome measurements, and (7) AEs. Discrepancies were resolved by consensus.

### Quality assessment

Two reviewers (Yang and Huang) independently evaluated the methodological quality of identified studies using the “risk of bias tool” under the domains of six aspects, including (1) sequence generation, (2) allocation concealment, (3) blinding, (4) incomplete outcome data, (5) selective outcome, and (6) other biases. The methodological criteria referred to the *Cochrane Handbook for Systematic Reviews of Interventions*, version 5.0.1 [24].

### Statistical methods

Results for dichotomous outcomes are expressed as risk ratios (RR) with 95 % CIs. Results for continuous outcomes are expressed as the mean difference (MD) (if the same scale for each trial was available) or standardized mean difference (if different scales were used). We evaluated heterogeneity among the included studies using the I^2^ test. We considered a value greater than 50 % to indicate substantial heterogeneity and sought the potential sources of heterogeneity (clinical heterogeneity and methodological heterogeneity). Regardless of the size of heterogeneity, the random effects model was used for statistical analysis. We conducted the meta-analysis using Cochrane RevMan 5.1. We planned to assess publication bias by following the recommendations on testing for funnel plot asymmetry according to the *Cochrane Handbook for Systematic Reviews on Interventions* [24]. We visually assessed funnel plot asymmetry.

## Results

### Results of the literature search

**Fig 1:**
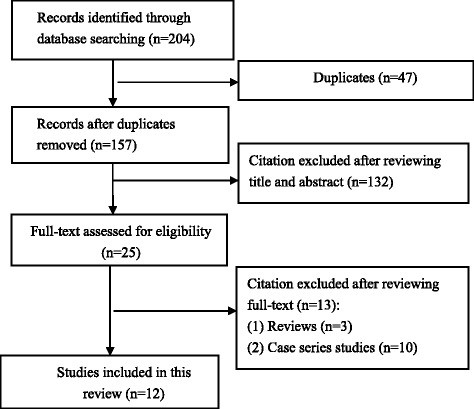
Flow chart of literature screening and the selection process

A total of 204 articles were retrieved from searching electronic databases and reference lists (Fig. 1). After removing duplicate articles and screening titles, abstracts, and full texts, 12 studies were included in this review.

### Characteristics of included studies

We included 12 studies involving 935 participants (710 were male). The sample size ranged from 48 to 195 cases. Participants were aged between 4 and 18 years. The proportion of male participants was 76 % (710/935). Nine studies were conducted in mainland China, two in Korea [10, 14], and one in Iran [9]. All of the studies used pharmacological interventions. Eleven studies were positive drug controls, which were defined as the control group having an active control (*i.e.*, test drug versus control drug, or standard treatment plus test drug versus standard treatment plus control drug). Of these 11 studies, seven studies used haloperidol as a control, three studies [25–27] used tiapride and one study [9] used risperidone. Only one study used placebo as a control [10]. For diagnostic criteria, six studies used the DSM-IV (four studies used the DSM-IV-Text Revision and two used the DSM-IV), three studies used the CCMD, and three studies used the ICD-10. Seven studies used the YGTSS, one study used the CGI Scale, and two studies used self-defined criteria by the authors for measurement of outcome. The period of treatment ranged from 8 to 12 weeks. See Table 1.

**Table 1:** General characteristics of included studies

Study	Characteristics of participants			Interventions		Treatment period (weeks)	Outcome measures indicators	Diagnostic criteria
	Age/Indication	Sample (male)	Comparability of baseline	Treatment group	Control group			
Ghanizadeh 2013 [9]	6-18 years;	60(49)	Comparable	Aripiprazole (initial dose: 1.25 mg/day, gradually increase dose, final dose: 10–15 mg/day)	Risperidone (initial dose: 0.25 mg, gradually increase dose, final dose: 2-3 mg/d)	8	1. YGTSS score	DSM-IV-TR
	Tic disorder						Baseline T*: 30.5 ± 13.4, C†: 31.7 ± 10.0	YGTSS ≥ 21
							After treatment T*: 12.8 ± 12, C†:19.3 ± 12.5	
							2. Motor tic severity score	
							Baseline T*: 13.1 ± 4.1, C†: 12.9 ± 3.8	
							After treatment T*: 4.0 ± 4.5, C†:6.0 ± 4.3	
							3. Vocal tic severity score	
							Baseline T*: 4.0 ± 4.1, C†: 6.1 ± 5.1	
							After treatment T*: 0.7 ± 1.8, C†:3.0 ± 3.8	
							4. Total tic severity scores	
							Baseline T*: 16.5 ± 6.4, C†: 19.0 ± 7.3	
							After treatment T*: 5.7 ± 6.2, C†:9.9 ± 7.7	
Yoo 2013 [10]	6-18 years Tourette syndrome	61(53)	Comparable	Aripiprazole (initial dose: 2 mg/day, gradually increase dose, maximum dose: 20 mg/day)	Placebo (initial dose: 2 mg/day, gradually increase dose, maximum dose: 20 mg/day)	9	1. Total tic severity scores	DSM-IV
							Baseline T*: 28.3 ± 5.5, C†: 29.5 ± 5.6	YGTSS ≥ 22
							After treatment T*: 13.6 ± 9.1, C†:19.9 ± 9.5	
							2. Motor tic severity score	
							Baseline T*: 15.9 ± 4.0, C†: 17.3 ± 3.2	
							After treatment T*: 8.6 ± 6.1, C†:11.9 ± 5.5	
							3. Vocal tic severity score	
							Baseline T*: 12.4 ± 3.7, C†: 12.2 ± 4.4	
							After treatment T*: 5.0 ± 4.6, C†:8.0 ± 5.5	
							4. Tourette’s syndrome clinical global	
							Baseline T*: 4.5 ± 0.8, C†: 4.7 ± 0.8	
							After treatment T*: 2.8 ± 1.4, C†:3.6 ± 1.3	
Yoo 2011 [14]	6-15 years; Tic disorder	48(33)	Comparable	Aripiprazole (initial dose: 5 mg/d, increments every 2 weeks: 5–10 mg/d, maximum dose: 20 mg/d)	Haloperidol (initial dose 0.75 mg/d and increased in 1.5–3 mg/day increments every 2 weeks, maximum dose: 4.5 mg/d	8	1. Total tic severity scores:	DSM-IV
							Baseline T*: 26.5 ± 4.9, C†: 27.6 ± 7.3	YGTSS ≥ 22
							After treatment T*:12.1 ± 6.4, C†: 10.1 ± 7.5.	
							2. Motor tic severity scores:	
							Baseline T*: 17.5 ± 5.3, C†: 20.5 ± 3.1	
							After treatment T*: 8.0 ± 4.4, C†: 8.5 ± 6.7	
							3. Vocal tic severity score:	
							Baseline T*: 9.0 ± 6.7, C†: 7.1 ± 8.3	
							After treatment T* 4.5 ± 4.6, C†: 2.4 ± 4.3	
Wang 2013 [25]	6-15 years; Tourette syndrome	60(41)	Unclear	Aripiprazole (initial dose: 2.5 mg/d, maximum dose: 10 mg/d)	Tiapride: (initial dose: 50 mg/d, maximum dose: 300 mg/d)	8	1. YGTSS score	ICD-10
							Baseline T*: 70.8 ± 9.9, C†: 70.1 ± 9.6	
							After treatment T*:44.5 ± 7.9, C†: 51.8 ± 8.3	
							2. Motor tic severity score	
							Baseline T*: 24.8 ± 6.5, C†: 23.1 ± 5.8	
							After treatment T*: 8.7 ± 6.3, C†: 9.5 ± 5.8	
							3. Vocal tic severity score	
							Baseline T*: 18.8 ± 8.4, C†: 18.8 ± 8.4	
							After treatment T*: 9.6 ± 7.8, C†: 11.5 ± 7.1	
							4. Impairment score:	
							Baseline T*: 31.4 ± 8.3, C†: 30.5 ± 8.8	
							After treatment T*:18.9 ± 7.8, C†: 22.4 ± 7.8	
Liu 2010 [26]	6-14 years; Tic disorder	65(57)	Comparable	Aripiprazole (initial dose:2.5 mg, qd, increase dose every week:	Tiapride (initial dose: 25 mg, bid, increase dose every week: 25 mg, maximum dose: 400 mg/d.	12	1. Author self-defined tics symptom improvement	DSM-IV-TR
							(Rate of progress in tics symptom ≥ 30 %)	
				2.5 mg, maximum dose:10 mg/d)				
							T*:91 %(30/33), C†: 84 %(26/31)	
							2. Decreased YGTSS score	
							T*: 64 ± 23, C†: 63 ± 25	
							3. Decreased motor tic severity score	
							T*: 68 ± 15, C†: 61 ± 15	
							4. Decreased vocal tic severity score	
							T*: 68 ± 15, C†: 61 ± 15	
							5. Decrease impairment score	
							T*: 59 ± 42, C†: 63 ± 48	
Liu 2011 [27]	5-17 years; Tourette syndrome	195(156)	Comparable	Aripiprazole (Age < 8 years: initial dose: 2.5 mg, qd, increase dose every week:2.5 mg, final dose 5-15 mg/d, qd. Age > 8 years:	Tiapride (Age < 8 years: initial dose: 25 mg, bid, increase dose every week: 50 mg, final dose 100-300 mg/d, bid or tid. Age > 8 years: initial dose 50 mg, bid, increase dose every week: 100 mg, final dose: 200-500 mg/d, bid or tid)	12	1. YGTSS score	DSM-IV-TR
							Baseline T*: 53.74 ± 15.71, C†: 51.66 ± 13.63	YGTSS ≥ 25
							After treatment T*: 24.36 ± 16.38,	
							C†: 23.26.1 ± 15.31	
				initial dose 5 mg, qd, increase dose every week: 5 mg, final dose: 10-25 mg/d, qd)			2. Motor tic severity score	
							Baseline T*: 15.93 ± 3.22, C†: 15.08 ± 2.97	
							After treatment T*: 7.69 ± 4.14, C†: 7.45 ± 3.42	
							3. Vocal tic severity score	
							Baseline T*: 11.99 ± 4.90, C†: 11.63 ± 3.88	
							After treatment T*: 4.19 ± 4.05, C†: 3.76.1 ± 3.57	
							4. Impairment score	
							Baseline T*: 25.71 ± 10.35, C†: 24.85 ± 9.37	
							After treatment T*: 12.45 ± 9.95, C†: 11.96.1 ± 9.86	
Cheng 2012 [28]	T*:8.1 ± 2.9	62(39)	Unclear	Aripiprazole (initial dose:2.5 mg, maximum dose: 10 mg/d)	Haloperidol (initial dose: 0.5 mg, maximum dose: 10 mg/d)	8	YGTSS score	CCMD-3
	C†:7.9 ± 3.2;						Baseline T*: 64.15 ± 15.52, C†: 66.34 ± 15.37	
	Tic disorder						After treatment T*: 17.59 ± 15.12,C†:25.05 ± 16.81	
Ren 2012 [29]	5-16 years; Tic disorder	68(58)	Comparable	Aripiprazole (initial dose: 2.5 mg/d, gradually increase dose, final dose: 5-20 mg/d)	Haloperidol (initial dose: 1 mg/d, gradually increase dose, final dose: 2-8 mg/d)	8	1. YGTSS score	DSM-IV-TR
							Baseline T*:55.32 ± 12.23, C†:54.56 ± 13.08	YGTSS ≥ 25
							After treatment T*: 21.52 ± 18.32, C†: 20.98 ± 16.45	
							2. Author self-defined tics symptom improvement	
							(Rate of progress in tics symptom ≥30 %)	
							T*:79 %(26/33), C†: 73 %(22/30)	
Zhao 2011 [30]	4-15 years; Tic disorder	108(72)	Comparable	Aripiprazole (initial dose: 5 mg, maintenance dose: 5-15 mg/d)	Haloperidol (initial dose:2 mg, maintenance dose: 2-12 mg/d)	8	CGI scale	CCMD-3
							T*:81.3 %(44/54), C†: 82.8 % (39/47)	
Guo 2013[31]	4-16 years; Tic disorder	80(55)	Comparable	Aripiprazole (initial dose:2.5 mg, maximum dose: 12.5 mg, average daily dose: 7.8 ± 1.1 mg)	Haloperidol (initial dose:1 mg, maximum dose: 16 mg, average daily dose:5.7 ± 0.8 mg)	8	YGTSS score	ICD-10
							Baseline T*: 65.43 ± 9.64, C†: 66.37 ± 10.16	YGTSS ≥ 25
							After treatment T*: 20.17 ± 10.32, C†: 19.87 ± 9.83	
Gao 2013 [32]	T*:11.2 ± 3.5;C†:8.6 ± 2.9; Tic disorder	48(33)	Comparable	Aripiprazole (initial dose:2.5 mg/d, increase dose every week: 2.5-5.0 mg/d, maximum dose: 20 mg/d)	Haloperidol (initial dose: 1 mg/d, increase dose every week: 2 mg/d, maximum dose: 8 mg/d.	8	1.Total tic severity score	CCMD-3
							Baseline T*: 26.5 ± 4.9, C†: 27.6 ± 7.3	YGTSS ≥ 22
							After treatment T*: 12.1 ± 6.4, C†: 10.1 ± 7.5	
							2. Motor tic severity score	
							Baseline T*: 17.5 ± 5.3, C†: 20.5 ± 3.1	
							After treatment T*: 8.0 ± 4.4, C†: 8.5 ± 6.7	
							3. Vocal tic severity score	
							Baseline T*: 9.0 ± 6.7, C†: 7.1 ± 8.3	
							After treatment T*: 4.5 ± 4.6, C†: 2.4 ± 4.3	
Liang 2010 [33]	4-16 years; Tourette syndrome	80(64)	Comparable	Aripiprazole (5–30 mg/d)	Haloperidol (6–16 mg/d)	8	YGTSS score	ICD-10
							Baseline T*: 54.95 ± 13.98, C†: 52.97 ± 13.54	YGTSS ≥ 25
							After treatment T*: 35.12 ± 13.83, C†:	
							19.26 ± 14.24	

Total Tic Severity Score = Motor Tic Severity score + Vocal Tic Severity score (0–50), Total Yale Global Tic Severity Scale Score = Total Tic Severity Score + Impairment score (0–100)

Decreased YGTSS score: (tics scores before treatment- tics scores after treatment) /tics scores before treatment

Rate of progress in tics symptom: (tics scores before treatment- tics scores after treatment) /tics scores before treatment

*CCMD* Chinese classification and diagnostic criteria of mental disorders; *DSM-IV* diagnostic and statistical manual of mental disorder-IV; *DSM-IV-TR* diagnostic and statistical manual of mental disorder-IV-Text Revision; *ICD-10* international code of diseases;

*: Treatment group; †: Control group

### Quality assessment

Twenty-five percent (3/12) of studies used an adequate method of random sequence generation [9, 29, 32]. One third (4/12) of the studies only mentioned “random allocation” without a specific description. Only one study [9] implemented adequate allocation concealment and blinding, three studies were open-label studies and did not use the method of blinding [14, 28, 33], and the remaining studies did not mention the details of blinding. Fifty percent (6/12) of studies reported loss to follow-up, and none of the studies used an intention-to-treat analysis for incomplete outcome data. One study stated that the reasons for loss to follow-up did not differ between two groups [10]. Only one of the studies had registration for a protocol [10]. Therefore, whether there was selective reporting was unclear. Comparability of baseline in two of the studies was unclear [25, 28]. In other trials, there were no significant differences in the comparability of baseline between the treatment group and the control group. See Table 2.

**Table 2:** Quality assessment of included studies

References	Quality assessment					
	Random sequence generation	Allocation concealment	Blinding	Incomplete outcome data	Selective reporting	Bias from other resources
Ghanizadeh2013 [9]	Low risk	Low risk	Low risk	Low risk	Unclear	Low risk
Yoo 2013 [10]	Unclear	Unclear	Unclear	Low risk	Low risk	Low risk
Yoo 2011 [14] ^a^	High risk	Unclear	High risk	Low risk	Unclear	Low risk
Wang 2013 [25]	High risk	Unclear	Unclear	Low risk	Unclear	High risk
Liu 2010 [26]	Unclear	Unclear	Unclear	Unclear	Unclear	Low risk
Liu 2011 [27]	High risk	Unclear	Unclear	Unclear	Unclear	Low risk
Cheng 2012 [28]	Unclear	Unclear	Unclear	Low risk	Unclear	High risk
Ren 2012 [29] ^a^	Low risk	Unclear	High risk	Unclear	Unclear	Low risk
Zhao 2011 [30]	High risk	Unclear	Unclear	Unclear	Unclear	Low risk
Guo 2013[31]	Unclear	Unclear	Unclear	Low risk	Unclear	Low risk
Gao 2013 [32] ^a^	Low risk	Unclear	High risk	Unclear	Unclear	Low risk
Liang 2010 [33]	High risk	Unclear	Unclear	Low risk	Unclear	Low risk

^a^: they are open-label studies which did not use the method of blinding, so we justify them as high risk. Many studies did not mention the details of blinding, so we justify them as unclear

### Analysis of efficacy and safety

#### Primary outcome measurements

##### Evaluation of efficacy by using the YGTSS

**Fig 2:**
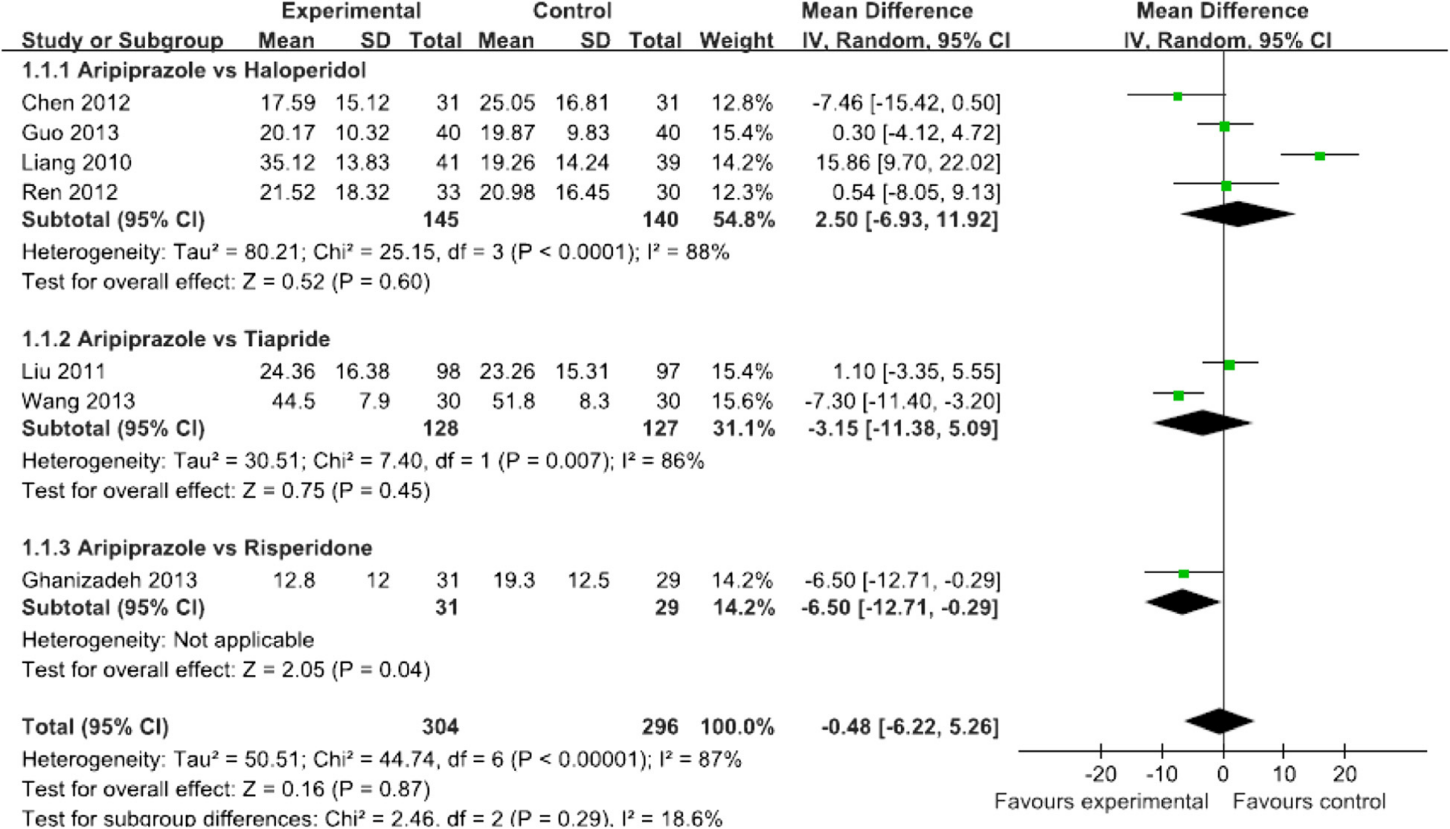
Meta-analysis of symptom improvement assessed by YGTSS Total Score

One randomized, double-blind, placebo-controlled study [10] used the total YGTSS score as the outcome measurement, and showed a significant difference in reduction of the total YGTSS score (13.6 ± 9.1 vs 19.9 ± 9.5, *P* < 0.05) and vocal tic score (5.0 ± 4.6 vs 8.0 ± 5.5, *P* < 0.05) between aripiprazole and placebo. There was no significant difference in reduction of the motor tic score (8.6 ± 6.1 vs 11.9 ± 5.5, *P* > 0.05). A total of seven studies (*N* = 600 patients) used the YGTSS scale as the outcome measurement, and there was no significant difference in reduction of the total YGTSS score between the aripiprazole and positive control groups (MD = −0.48, 95 % CI [−6.22, 5.26], *P* = 0.87, I^2^ = 87 %) (Fig. 2) during the treatment period. Of them, meta-analysis of four of the studies (*N* = 285 patients) that compared aripiprazole with haloperidol showed that there was no significant difference in reduction of the total YGTSS score between the two groups (MD = 2.50, 95 % CI [−6.93, 11.92], *P* = 0.60, I^2^ = 88 %) [28, 29, 31, 33]. Meta-analysis of two studies (*N* = 255 patients) that compared aripiprazole with tiapride showed that there was no significant difference in reduction of the total YGTSS score between the two groups (MD = −3.15, 95 % CI [−11.38, 5.09], *P* = 0.45, I^2^ = 86 %) [25, 27]. One study (*N* = 60 patients) showed that there was a significant difference in reduction of the total YGTSS score between aripiprazole and risperidone (12.8 ± 12 vs 19.3 ± 12.5, *P* < 0.001), but aripiprazole was not superior to risperidone for improving the YGTSS (MD = −6.50, 95 % CI [−12.71, −0.29], *P* = 0.04) [9].

**Fig 3:**
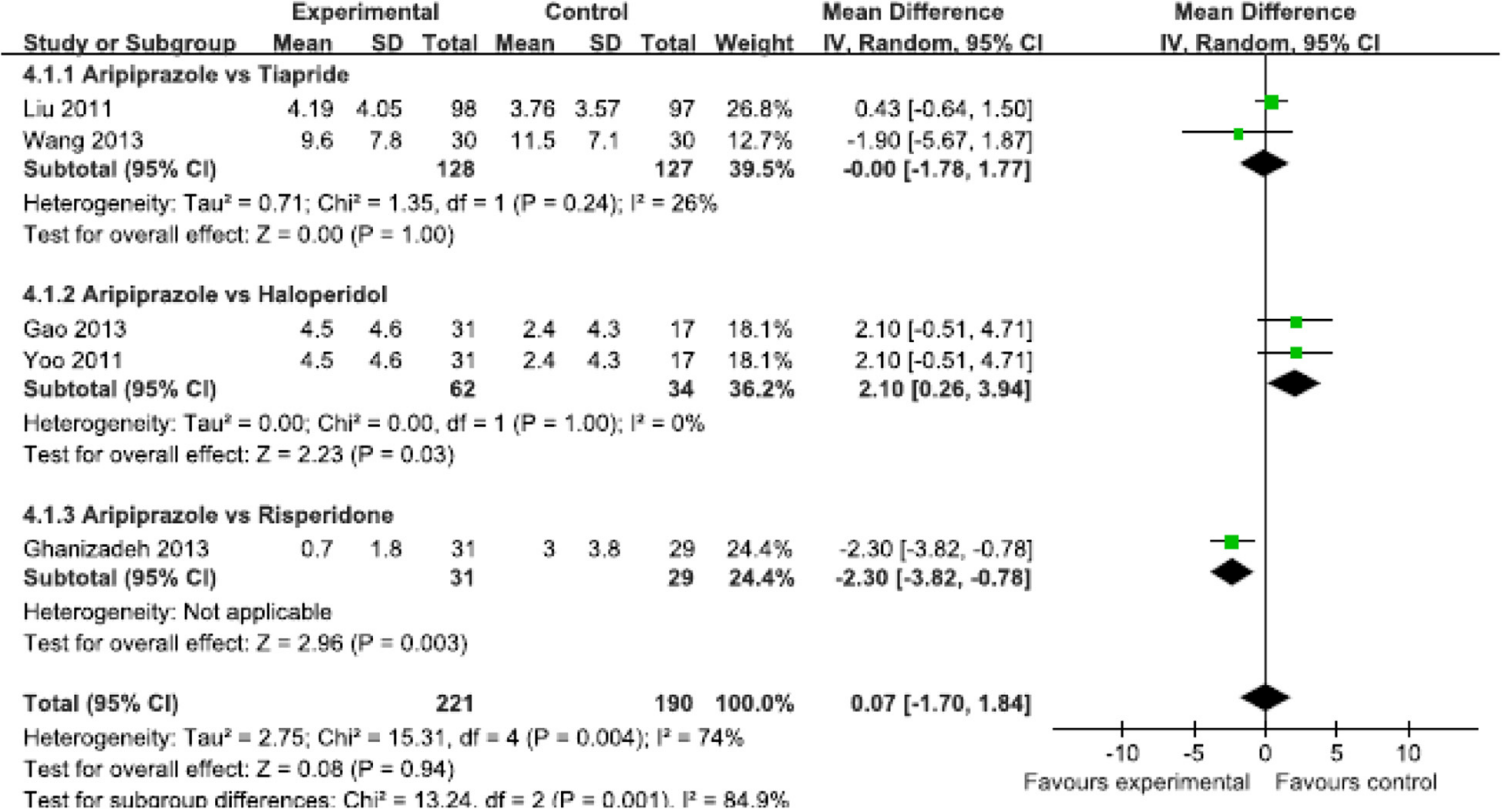
Meta-analysis of symptom improvement assessed by YGTSS Vocal Tics Score

**Fig 4:**
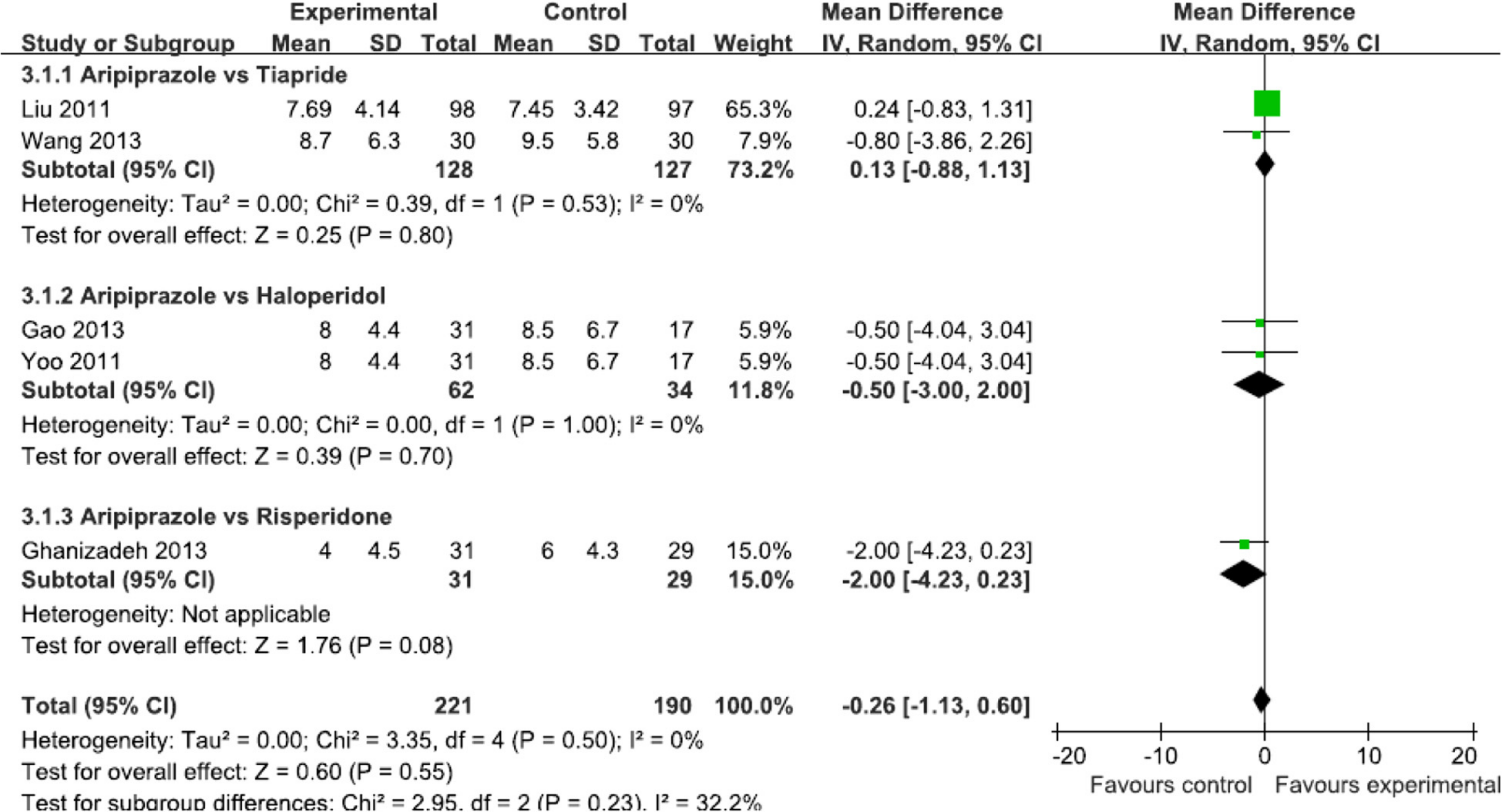
Meta-analysis of symptom improvement assessed by YGTSS Motor Tics Score

**Fig 5:**
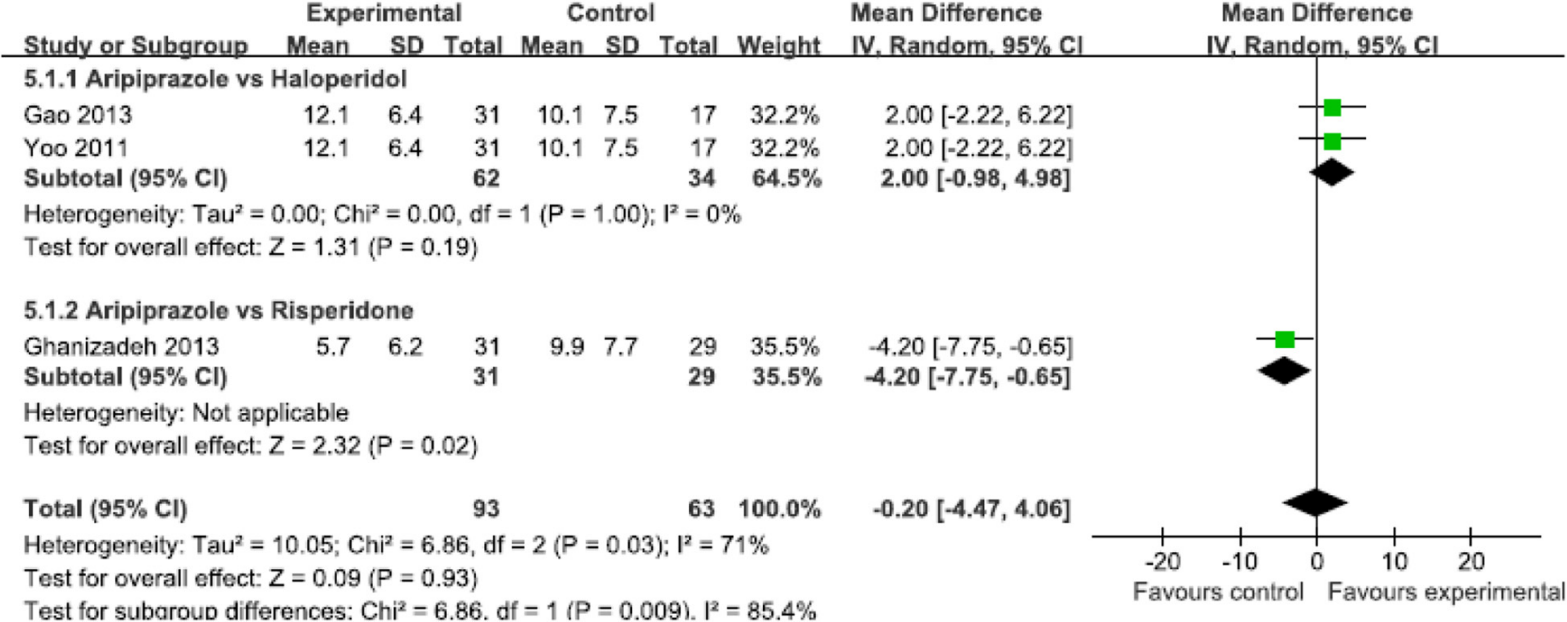
Meta-analysis of symptom improvement assessed by YGTSS Total Tics Score

**Fig 6:**
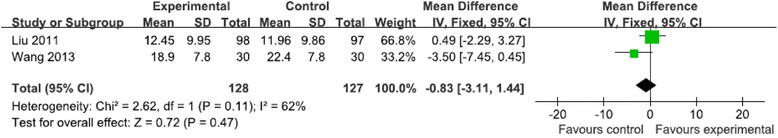
Meta-analysis of symptom improvement assessed by YGTSS Impairment Score

Five studies (*N* = 411 patients) evaluated motor and vocal tics, and showed no significant difference in reduction of the vocal tics score (MD = 0.07, 95 % CI [−1.70, 1.84], *P* = 0.94, I^2^ = 74 %) (Fig. 3) and the motor tics score (MD = −0.26, 95 % CI [−1.13, 0.60], *P* = 0.55, I^2^ = 0) (Fig. 4) between the aripiprazole and positive control groups. Three studies (*N* = 156 patients) evaluated the total tic score, and showed no significant difference in reduction of the total tic score (MD = −0.2, 95 % CI [−4.47, 4.06], *P* = 0.93, I^2^ = 71 %) (Fig. 5). Two studies (*N* = 255 patients) evaluated the impairment score and also showed no significant difference in reduction of the impairment score (MD = −0.83, 95 % CI [−3.11, 1.44], *P* = 0.47, I^2^ = 62 %) (Fig. 6).

##### Evaluation of efficacy by using the CGI Scale

One study used the CGI Scale as the outcome measure, and showed no significant difference in the rate of clinical efficacy between aripiprazole and haloperidol (81.3 % [44/54] vs 82.8 % [39/47], *P* > 0.05) [30].

##### Evaluation of efficacy by using the Tourette Syndrome Global Scale

One study used the Tourette Syndrome Global Scale as the outcome measure, and showed a significant difference in the Tourette Syndrome Global Scale between aripiprazole and placebo (MD = −0.80, 95 % CI [−1.48, −0.12], *P* = 0.02) [10].

#### Secondary outcome measurements

##### Efficacy of improvement of tic symptoms that were self-defined by authors

**Fig 7:**
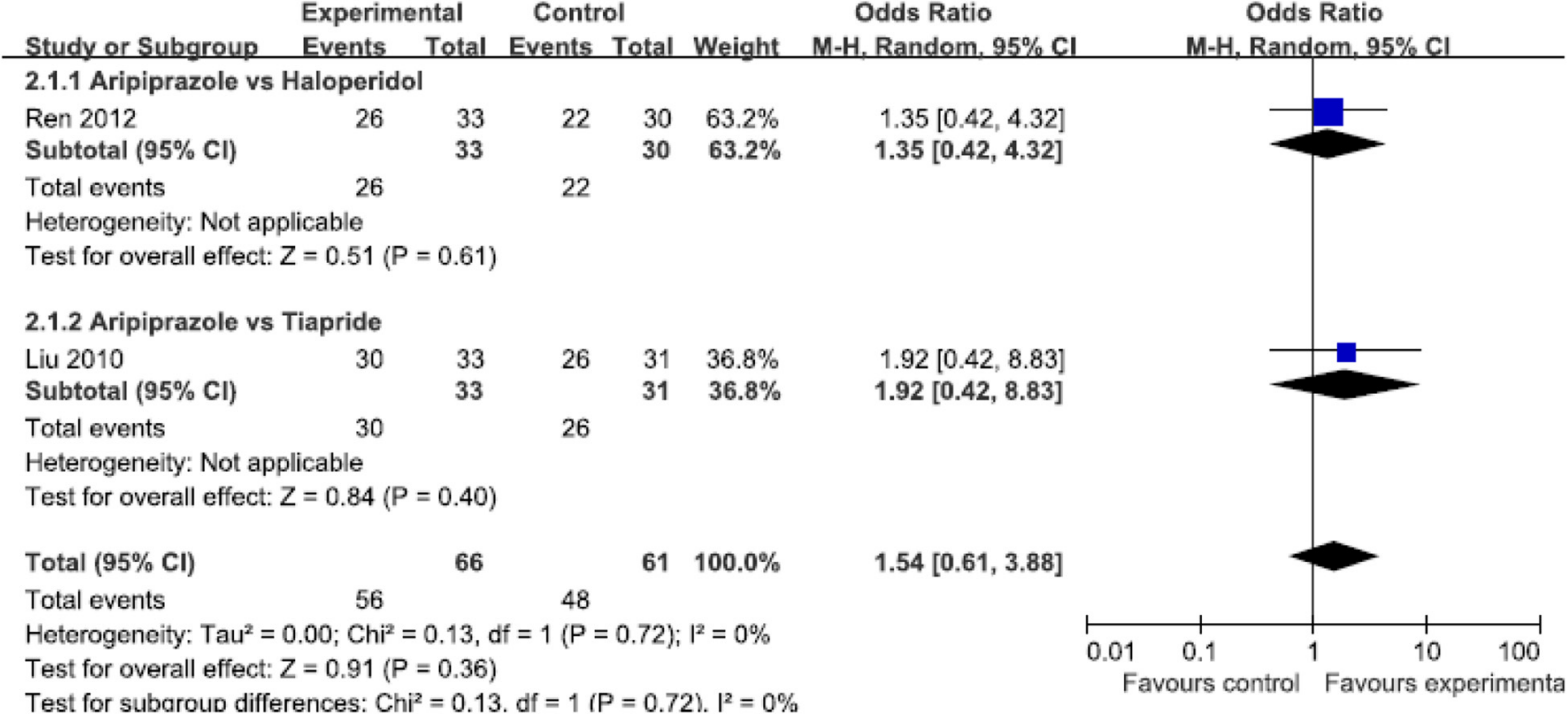
Meta-analysis of tics symptom improvement assessed by tics symptom improvement by author self-defined

Meta-analysis of two studies (*N* = 127 patients) used the rate of progress in tic symptoms ≥30 % as the outcome measure. There was no significant difference between the aripiprazole and positive control groups (RR = 1.54, 95 % CI [0.61, 3.88], *P* = 0.36, I^2^ = 0) [29, 31]. See Fig. 7.

#### AEs

All of the studies reported specific AEs, except for one study [30]. The most common AEs of aripiprazole were drowsiness and increased appetite. The most common AEs of haloperidol and tiapride were drowsiness, extrapyramidal symptoms, nausea, and dizziness. The most common AEs of risperidone were increased appetite, drowsiness, and diurnal urinary incontinency. The most common AEs of placebo were dizziness, akathisia and sedation. The common AEs of different drugs are shown in Table 3.

**Table 3:** The reported AEs of included studies

System	Aripiprazole	Haloperidol	Tiapride	Risperidone
Neuromuscular system and mental symptom	Drowsiness:5.1 %(5/98)–58.1 %(18/31);	Drowsiness: 6.67 %(2/30)–82.4 %(14/17);	Dizziness: 3.1 %(3/97)–6.67 %(2/30);	Drowsiness:17.2 %(5/29);
	Extrapyramidal symptoms: 6.45 %(2/33)–19.4 %(6/31);	Extrapyramidal symptoms: 40 %(12/30)–43.6 %(17/39);	Drowsiness: 3.23 %(1/31)–5.2 %(5/97);	Fatigue: 3.4 %(1/29);
	Headache: 2 %(2/98)–16.1 %(5/31);	Tremor: 19.4 %(6/31)–22.5 %(9/40);	Anxiety: 13.3 %(4/30);	Dizziness: 3.4 %(1/29);
	Akathisia:3.33 %(1/30)–6.3 %(2/32);	Headache: 58.8 %(10/17);	Sedation: 6.67 %(2/30);	Nausea: 3.4 %(1/29);
	Anxiety: 2 %(2/98)–6.45 %(2/31); Tremor: 3.23 %(1/31)–5 %(2/40);	Dizziness: 11.8 %(2/17);	Akathisia: 6.67 %(2/30);	
	Fatigue: 2 %(2/98)–9.7 %(3/31);	Emotional hypersensitivity: 11.8 %(2/17);	Fatigue: 3.1 %(3/97);	
	Dizziness: 2.44 %(1/41)–6.5 %(2/31);	Insomnia: 11.8 %(2/17); Irritability:11.8 %(2/17); Fatigue: 7.69 %(3/39); Nightmare: 5.9 %(1/17);	Headache: 2.1 %(2/97);	
	Insomnia: 1 %(1/98)–3.2 %(1/31); Sedation:12.5 %(4/32);		Insomnia : 2.1 %(2/97);	
	Slowness: 6.5 %(2/31); Tiredness: 4.88 %(2/41);			
	Emotional hypersensitivity: 3.2 %(1/31); Irritability: 3.2 % (1/31);			
	Nightmare: 3.2 %(1/31)			
Digestive system	Increased appetite: 3.2 %(1/31)–25.8 %(8/31);	Nausea/vomiting: 23.5 %(4/17);	Nausea: 3.1 %(3/97)–13.3 %(4/30);	Increased appetite:
	Anorexia: 4.1 %(4/98)–15 %(6/40); Nausea: 2 %(2/98)–18.8 %(6/32);	Nausea: 16.1 %(5/31);	Anorexia: 3.23 %(1/31)–4.1 %(4/97);	27.6 %(8/29);
	Nausea/vomiting:1 %(1/98)–29 %(9/31);	Gastrointestinal disturbances: 11.8 %(2/17);	Nausea/vomiting: 2.1 %(2/97);	Abdominal pain: 6.9 %(2/29);
	Decreased Appetite: 12.9 %(4/31); Abdominal pain 9.7 %(3/31);	Anorexia: 7.5 %(3/40)–11.8 %(2/17);		
	Gastrointestinal disturbances: 6.5 %(2/31); Dyspepsia: 3.1 %(1/32);	Constipation: 6.45 %(2/31);		
	Abnormal liver function: 1 %(1/98);	Increased appetite: 5.9 %(1/17)		
Ocular region	Blurred vision: 3.2 %(1/31)–9.7 % (3/31)	-	-	Blurred vision: 10.3 %(3/29);
Endocrine system	Weight gain: 1 %(1/98); Polydipsia: 3.2 %(1/31)	-	-	-
Urinary system	Nocturia: 3.2 %(1/31)	Nocturia: 5.9 %(1/17);	Nocturia: 1 %(1/97)–3.23 %(1/31);	Diurnal Urinary incontinency: 13.8 %(4/29);
Cardiovascular system	Elecrocardiogram QT prolonged: 6.3 %(2/32);	Electrocardiographic abnormality: 6.45 %(2/31)–10 %(4/40);	-	-
	Electrocardiographic abnormality: 2.5 %(1/40)–6.45 %(2/31);	Chest discomfort: 11.8 %(2/17)		
	Chest discomfort: 3.2 % (1/31)			
Respiratory system	Nasopharyngitis: 12.5 %(4/32);	-	-	-
	Upper respiratory tract infection: 3.1 %(1/32);			
Skin	Itches: 3.2 %(1/31);	-	-	Itches: 10.3 %(3/29);
Others	Dry mouth: 6.5 %(2/31)–6.67 %(2/30);	Dry mouth: 5 %(2/40)–19.4 %(6/31);	Dry mouth: 10 %(3/30);	-
		Tiredness: 15.4 %(6/39); Joint pain: 11.8 %(2/17);		
		Febrile sense: 5.9 %(1/17);		
		School refusal: 5.9 %(1/17);		

#### Publication bias

**Fig 8:**
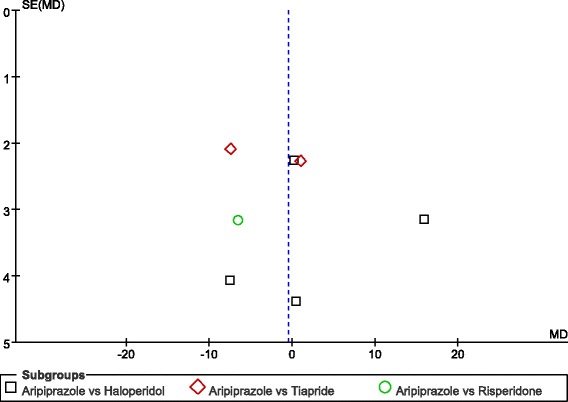
Funnel plot asymmetry for the included studies which using YGTSS Scale for efficacy evaluation

In this study, we visually assessed funnel plot asymmetry for the included studies that used the YGTSS for evaluation of efficacy. Figure 8 shows that the funnel plot was asymmetric.

## Discussion

In this systematic review, we analyzed the efficacy and safety of aripiprazole for children with TDs, we found similar findings of efficacy among a variety of studies that compared aripiprazole and an active agent or placebo. Some studies showed that aripiprazole improved the YGTSS scores in patients with TD. Although the quality of some studies was generally poor, at least they were safe because there were no severe AEs. In general, aripiprazole was well tolerated. The most common AEs of aripiprazole were drowsiness, increased appetite, nausea, and headache. Tardive dyskinesia was observed in the typical antipsychotics (*i.e.*, haloperidol and pimozide). To minimize such side effects, atypical antipsychotics have been prescribed for patients with TDs as alternatives. Because of a lack of long-term evaluation of outcomes, some AEs was not observed in current studies. Long-term side effects of aripiprazole should be monitored in further studies.

The quality of the included studies in our review was generally poor. The main problems are as follows. Most studies were often labeled as “random” without providing details on random sequence generation. Only one study implemented adequate allocation concealment and blinding. Successful implementation of an adequately concealed randomization sequence and blinding were not reported. Most studies used a positive drug control as a control group, with a lack of placebo controls.

Seven studies used the YGTSS as the outcome measurement, but we found large heterogeneity in the meta-analysis. The potential reasons for this large heterogeneity may be as follows. (1) Only participants with a YGTSS score >25 points were included in four studies [27, 29, 31, 33, 35], the YGTSS score was >22 points in three studies [10, 14, 32], and the YGTSS score was >21 in one study [9]. (2) There were different diagnostic criteria that were used between studies. Three studies used the DSM-IV-Text Revision [9, 27, 29], three studies used the ICD-10 [25, 31, 33], and one study used the CCMD-3 [28]. Patients in three of the studies [25, 27, 33] were diagnosed with TS and the other patients had TDs. (3) There were differences in intervention and dose between studies. Four studies used haloperidol as a control, two studies used tiapride, and one study used risperidone. The initial and maximum dose varied in these included studies.

TS is defined by the onset of motor and vocal tics in children, lasting more than 12 months. Although TS is the most notorious cause of chronic tics, there are types of TDs that are more common in children than in adults. According to the DSM-IV of the American Psychiatric Association, other TDs include chronic motor TD and chronic vocal TD, which are defined as having motor or phonic tics (but not both) for more than 12 months. TTD is characterized by tics (either motor and/or vocal) for a duration of less than 12 months [35]. TS is often associated with behavioral problems, such as ADHD and obsessive-compulsive disorder [35], and is more difficult to treat than other TDs. In our study, four studies evaluated the efficacy of aripiprazole for TS, and in all of them, the YGTSS score was significantly reduced from baseline to after treatment. Therefore, the efficacy of aripiprazole for TS is promising.

There are several limitations to our study. (1) Most of the included studies were conducted in a single center with a small sample (48–195 cases), and were conducted in China. Therefore, the efficacy of aripiprazole needs to be tested in other ethnicities. (2) The outcome measurements varied across different studies, which made it difficult to compare the efficacy among different studies. (3) The majority of studies used positive drugs as controls, with a lack of reasonable placebo controls. (4) There was a lack of long-term evaluation of outcomes in the included studies. AEs, such as metabolic side effects and tardive dyskinesia, were not observed in current studies. Long-term side effects of aripiprazole need to be monitored in further studies. (5) Monitoring the quality of implications and reporting of trials was difficult because of the lack of clinical trial registration, and publication bias may exist.

## Conclusions

In conclusion, aripiprazole appears to be a promising therapy for children with TDs. Further well-conducted RCTs are required to confirm this issue.
